# 2147. Design of a Ligand-Targeted Immunotherapy for Treatment of Influenza Virus Infections

**DOI:** 10.1093/ofid/ofad500.1770

**Published:** 2023-11-27

**Authors:** Imrul Shahriar, Charity L Campbell, Ananda K Kanduluru, Madduri Srinivasarao, Philip S Low

**Affiliations:** Purdue University, West Lafayette, Indiana; Purdue University, West Lafayette, Indiana; Eradivir Inc., West Lafayette, Indiana; Eradivir Inc., West Lafayette, Indiana; Purdue University, West Lafayette, Indiana

## Abstract

**Background:**

Nearly 10% of world’s population is affected by influenza virus infections and more than 26 million Americans were infected during the 2022-2023 flu season, with 290,000-640,000 patients requiring hospitalization. While flu vaccines are widely available, they were only 36% effective during the 2021-2022 season. Standard-of-care (SOC) drugs such as Tamiflu and Xofluza are only effective when taken in the early stages of infection requiring novel flu drugs for treating later stages of infection.

Schematics of the mechanism of action of EV21

**Figure:**
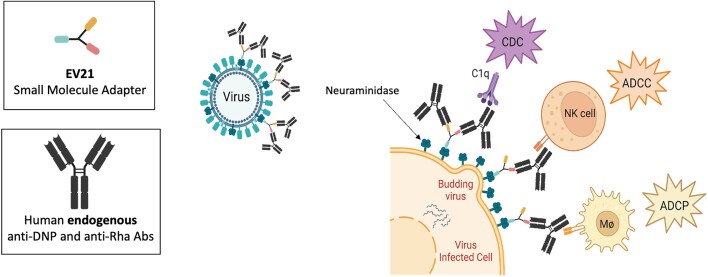

**Methods:**

Herein we report a targeted therapy (EV21) with a dual mechanism of action that elicits a host immune response to infections. Because neuraminidase is expressed on both the viral envelope and infected host cell surface, we repurposed the neuraminidase inhibitor zanamivir for use as the targeting ligand. We linked zanamivir to two distinct haptens that bind to two different naturally occurring antibodies in humans. Once recruited, these antibodies recruit the innate immune system to kill both virus and virus-infected cells. Preclinical in vitro and in vivo experiments were performed to evaluate the efficacy, safety, and pharmacokinetics of EV21.

**Results:**

When tested in BALB/c mice supplemented with physiological levels of human IgG (IVIG) and infected with 10xLD50 of influenza A virus (H1N1, A/Puerto Rico/8/1934), EV21 elicited excellent antiviral activity. A single dose of our EV21 showed much better activity in late-stage infections than daily doses of the SOC drugs. Also, EV21 caused a significantly faster reduction of viral titer in the lungs of infected mice. Although EV21 exerts its antiviral effect by engaging the innate immune system, it did not give rise to any local or systemic cytokine storm while protecting mice from virus-induced cytokine release syndrome. Moreover, EV21 demonstrated excellent safety, pharmacokinetics, and metabolic stability making it a promising clinical candidate.

**Conclusion:**

Our zanamivir-targeted dual hapten immunotherapy (EV21) has the potential to treat both early and late-stage influenza infections more effectively and rapidly than SOC drugs. Further testing in more clinically relevant ferret models and against multiple influenza strains, however, will be necessary to confirm the above benefits.

**Disclosures:**

**Imrul Shahriar, PhD Candidate**, Eradivir Inc.: Ownership Interest|Eradivir Inc.: Stocks/Bonds **Charity L. Campbell, n/a**, Eradivir Inc.: Salary **Ananda K. Kanduluru, PhD**, Eradivir Inc.: Salary|Eradivir Inc.: Ownership Interest|Eradivir Inc.: Stocks/Bonds **Madduri Srinivasarao, PhD**, Eradivir Inc.: Ownership Interest|Eradivir Inc.: Stocks/Bonds **Philip S. Low, PhD**, Eradivir Inc.: Board Member|Eradivir Inc.: Ownership Interest|Eradivir Inc.: Stocks/Bonds

